# Effects of Lignans from *Schisandra chinensis* Rattan Stems against Aβ_1-42_-Induced Memory Impairment in Rats and Neurotoxicity in Primary Neuronal Cells

**DOI:** 10.3390/molecules23040870

**Published:** 2018-04-10

**Authors:** Bing-You Yang, Wei Han, Hua Han, Yan Liu, Wei Guan, Xiao-Mao Li, Hai-Xue Kuang

**Affiliations:** Key Laboratory of Chinese Materia Medica, Ministry of Education of Heilongjiang University of Chinese Medicine, Harbin 150040, China; ybywater@163.com (B.-Y.Y.); hljhanwei1988@163.com (W.H.); hh7551@163.com (H.H.); lifeliuyan@163.com (Y.L.); myguanwei1234@yeah.net (W.G.); 18246074841@163.com (X.-M.L.)

**Keywords:** Magnoliaceae, Alzheimer’s disease, oxidative stress, apoptosis, cognitive ability

## Abstract

Oxidative stress, which is caused by Amyloid-β deposition in brain, plays an important role in Alzheimer’s disease. In this study, we found that lignans from *Schisandra chinensis* rattan stems (rsSCH-L) could reduce the escape latency and the distance travelled by the Aβ_1–42_ injected rats while the crossing platform time was enhanced in the Morris water maze test. Further research demonstrated that lignans from rsSCH-L attenuated Aβ_1-42_-induced neuronal cell injury by increasing the content of SOD and GSH-Px and decreasing the levels of LDH, ROS, and MDA. Moreover, rsSCH-L also inhibited the apoptosis of primary neuronal cells. The mechanisms of the apoptosis were related with the downregulation of caspase-3, caspase-8, Bax, and upregulation of Bcl-2. Taken together, the results show that rsSCH-L can improve cognitive ability in vivo. Meanwhile rsSCH-L exhibit a neuroprotective environment against oxidative stress and apoptosis in vitro. Therefore, rsSCH-L may be a potential therapeutic agent for this neurodegenerative disease.

## 1. Introduction

Alzheimer’s disease (AD), which is characterized as a loss of neurons, a loss of Aβ deposits, and formation of senile plaques, is a common neurological disease [1,2]. The clinical manifestations of AD include progressive memory loss, cognitive disorder, behavioral disorder, loss of self-care ability, severe dementia to a vegetative state, and, ultimately, death [3,4]. Although many hypotheses about the cause of this disease has proven that oxidative damage induced by Aβ toxicity is the main potential mechanism of the disease [5,6]. In the AD brain, the destroyed original oxidative stress balance between the reactive oxygen species and antioxidant defenses, which cause the disfunction of protein, DNA, lipid membranes, mitochondria, cellular function disruption, and integrity [7,8,9,10]. Neuronal cells play a key role in cognitive functions [11]. It has been proven that oxidative stress has a damaging effect on neuronal cells and can induce apoptosis in cells [12]. Therefore, antioxidants can alleviate the damage of oxidative stress to nerve cells and improve the cognitive and memory ability of neurodegenerative diseases [13].

*Schisandra chinensis* Turcz. Baill. (*S. chinensis*) is a traditional Chinese herbal medicine, which belongs to the Magnoliaceae family and the Schisandra genus [14,15,16]. Previous studies have been reported that the fruit extracts of *S. chinensis* possess the abilities of anti-Alzheimer’s disease and neuroprotective effect [11,17,18,19]. The research of our group showed that the rattan stems can improve the cognitive impairment and protect neuronal cells as well [20,21]. The rattan stems are 25.9 times as heavy as the fruits in the whole dry plant. The lignans are at maximum levels in the rattan stems [22,23,24]. The chemical compositions in *S. chinensis* rattan stems are similar to those in fruits such as lignans [25,26], monoterpene glycosides [27], and triterpenoids [28]. Lignans in fruits are considered major bioactive constituents [26,29,30]. Therefore, rattan stems may be potential alternatives in the treatment of AD.

For these reasons, we made a decision to explore the potential improvement effect of the lignans from *S. chinensis* rattan stems (rsSCH-L) against Aβ_1-42_-induced cognitive impairment and the mechanisms of neuroprotective effect.

## 2. Results

### 2.1. Effects of rsSCH-L on the Morris Water Maze Test in Aβ_1-42_-Induced AD Model Rat

**Figure 1:**
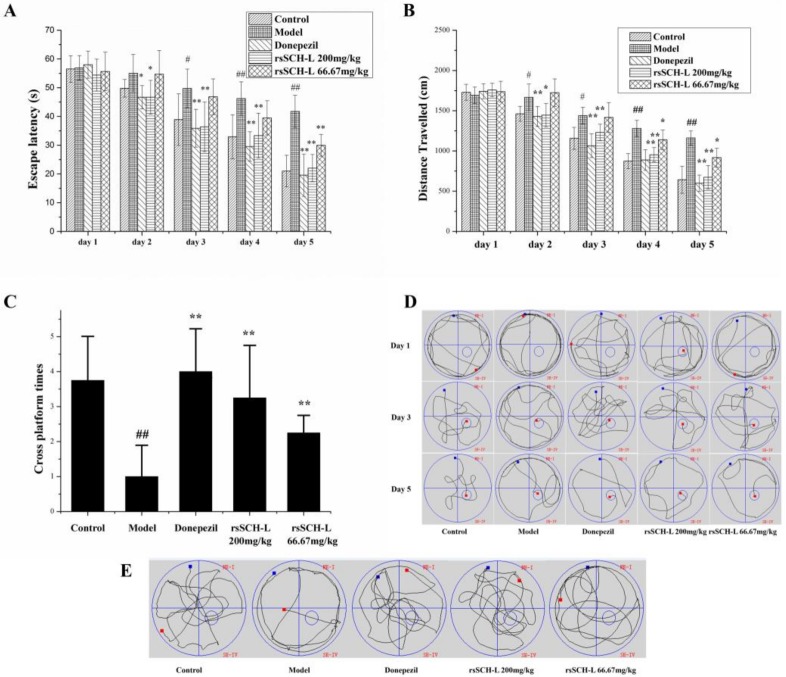
Effects of rsSCH-L on Aβ_1-42_-induced cognitive impairment in rats. (*n* = 10) (**A**) Escape latency, (**B**) Distance travelled, (**C**) Cross platform times, (**D**) Representative search strategy of rats in the trial on the first, third, and fifth days, (**E**) Representative swim paths during the spatial probe test are shown. ^#^ *p* < 0.05, ^##^ *p* < 0.01 vs control group, * *p* < 0.05, ** *p* < 0.01 vs. Aβ_1-42_ group.

As shown in Figure 1A,B, the escape latencies and the total distance travelled of all groups decreased along with the increment of the day. During the last three training days, it took a long time for the rats in the model group to reach the platform (see Figure 1D). Compared with the model group, the rsSCH-L 200 mg/kg group obviously ameliorated the effects of Aβ_1–42_ on escape latency and distance travelled. The latencies of the rsSCH-L 66.67 mg/kg group were lower than the model group on the last day. Distance travel had a difference in the last two days. The Donepezil group could decrease escape latency and distance travelled compared with the model group. On the 30th day, the platform was removed from the pool. The crossing platform location was recorded. The cross platform times of the rats’ treatment with rsSCH-L were more than that of the model group (see Figure 1C). The trajectory of searching for the hidden platform was also recorded (see Figure 1E). Together, these results suggested that rsSCH-L treatment could diminish the Aβ_1-42_-induced impairments of learning and memory.

### 2.2. Immunofluorescence Identification of Neuronal Cells

**Figure 2:**
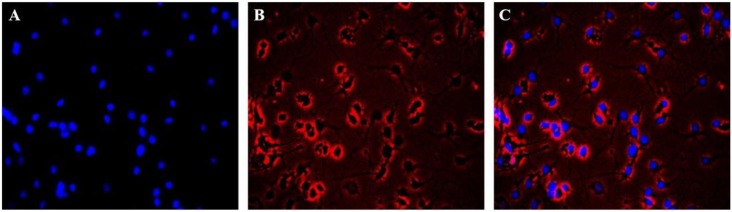
Primary cultured cortical neuronal cells were identified by β-III Tubulin. (**A**) The nucleus was stained with Hoechst 33258. (**B**) Immunocytochemical signals (red) under fluorescence microscope. (**C**) A merged image (**A** and **B**).

The immunofluorescence results of Hoechst 33258 and β-III Tubulin indicated the equal distribution of neuronal cells. The cells stained with Hoechst 33258 showed blue fluorescence in the nucleus (see Figure 2A) and the cells were red fluorescence by β-III Tublin (see Figure 1B). The results showed that the neurons were evenly distributed in the whole field, but only a few of the nuclei showed no red fluorescence (see Figure 2C). The purity of the neuronal cells was greater than 95% by β-III Tublin immunofluorescence identification.

### 2.3. Effects of rsSCH-L on Cell Viability and LDH Release in Aβ_1-42_-Induced Neuronal Cells

**Figure 3:**
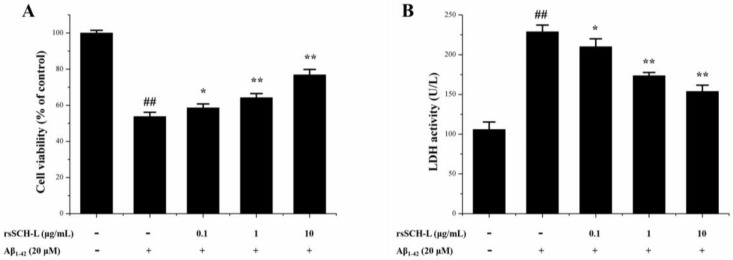
Effects of rsSCH-L on cell viability and LDH activity induced by Aβ_1-42_ in neuronal cells. (**A**) MTT assay of neuronal cells exposed to Aβ_1-42_ injury or treatment with rsSCH-L (*n* = 3), and (**B**) LDH release in the cell supernatant. (*n* = 3) ^##^ *p* < 0.01 vs. control group, * *p* < 0.05, ** *p* < 0.01 vs. Aβ_1-42_ group.

MTT and LDH were used to evaluate the effect of rsSCH-L on Aβ_1-42_ induced primary neuronal cells. Compared with the control group, the cell viability was decreased when induced by Aβ_1-42_. After treatment with rsSCH-L, the viability of the cells was significantly increased in concentration-dependent effects (see Figure 3A).

The degree of the cell membrane damage was measured by the level of LDH activity in supernatant. The LDH activity increased when induced by Aβ_1-42_. After treated with rsSCH-L, LHD activity was reduced. These findings indicated that rsSCH-L could protect neuronal cells from injury and attenuate the cytotoxicity induced by Aβ_1-42_ (see Figure 3B).

### 2.4. Effects of rsSCH-L on the Levels of ROS, MDA, and Activity of SOD and GSH-Px Release in Aβ_1-42_-Induced Neuronal Cells

**Figure 4:**
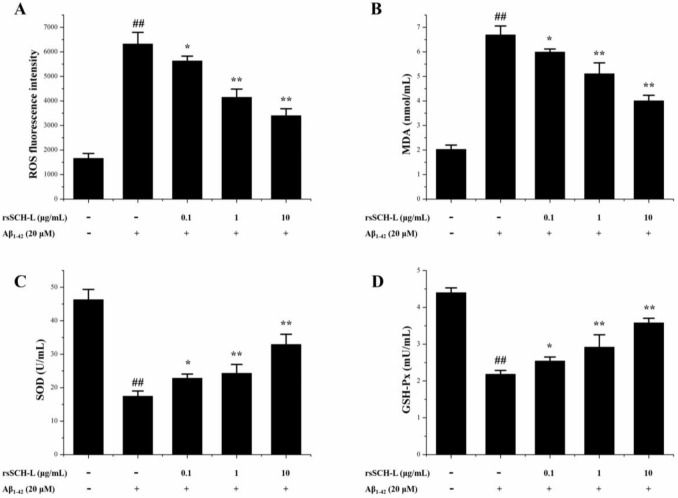
Effects of rsSCH-L in Aβ_1-42_-induced neuronal cells on the levels of ROS and MDA and activity of SOD and GSH-Px release. (**A**) Effect of rsSCH-L on the ROS level in neuronal cells. (*n* = 3); (**B**) Effect of rsSCH-L on the MDA level in neuronal cells. (*n* = 3); (**C**) Effect of rsSCH-L on the SOD activity in neuronal cells. (*n* = 3); (**D**) Effect of rsSCH-L on the GSH-Px activity in neuronal cells. (*n* = 3) ^##^ *p* < 0.01 vs. control group, * *p* < 0.05, ** *p* < 0.01 vs. Aβ_1-42_ group.

The antioxidant effects of rsSCH-L were detected in cells by using the DCFH-DA assay, the hydroxyl radical scavenging assay, the superoxide anion scavenging assay, and the peroxide metabolites scavenging assay [8,31]. Compared with the control group, the levels of ROS and MDA in the Aβ_1-42_ group were markedly increased. After treatment with rsSCH-L, the levels of ROS (see Figure 4A) and MDA (see Figure 4B) decreased, respectively. 

As shown in Figure 4C,D, Aβ_1-42_ significantly decreased SOD and GSH-Px activities in the cells. After incubation with rsSCH-L, the activities of SOD and GSH-Px were significantly increased when compared with the Aβ_1-42_ group.

### 2.5. Effects of rsSCH-L on Apoptosis in Aβ_1-42_-Induced Neuronal Cells

**Figure 5:**
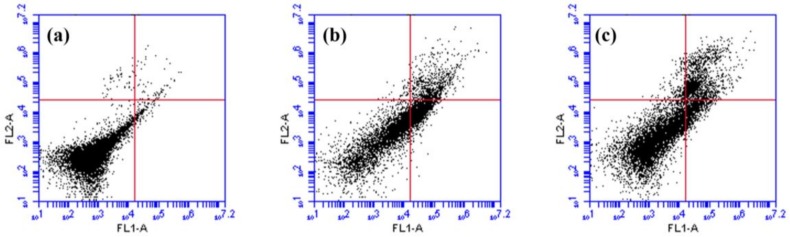

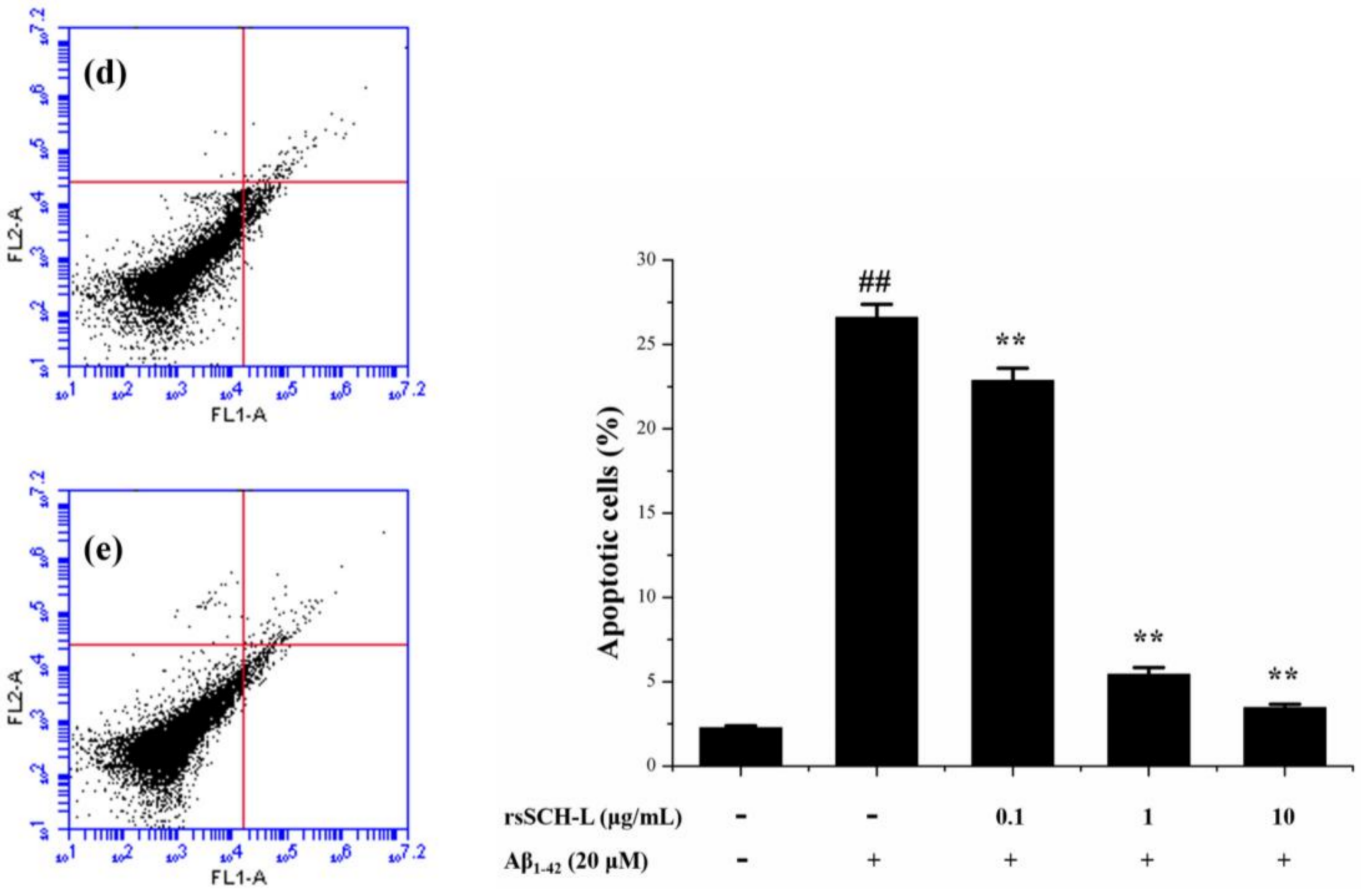
Effects of rsSCH-L on Aβ_1-42_-induced neuronal cell apoptosis, which was detected by flow cytometry. (*n* = 3); (**A**) Control group, (**B**) Aβ_1-42_ group; (**C**) Aβ_1-42_ + rsSCH-L (0.1 μg/mL) group; (**D**) Aβ_1-42_ + rsSCH-L (1 μg/mL) group, and (**E**) Aβ_1-42_ + rsSCH-L (10 μg/mL) group. Similar results were obtained from three independent experiments. ^##^ *p* < 0.01 vs. control group, ** *p* < 0.01 vs. Aβ_1-42_ group.

The effect of rsSCH-L on neuronal cell apoptosis was examined to test the neuro-protectant of rsSCH-L. As shown in Figure 5, after incubating with Aβ_1-42_ for 24 h, the total percentage of apoptotic cells (early + late apoptosis) was significantly increased from 2.3% to 26.6%. However, treatment with 0.1, 1, and 10 μg/mL rsSCH-L, the total percentage of apoptotic cells was 22.9%, 5.4%, and 3.5%, respectively. rsSCH-L markedly inhibited cell apoptosis.

### 2.6. Effects of rsSCH-L Regulated Caspase-3, Caspase-8, Bax, and Bcl-2 Levels in Aβ_1-42_-Induced Neuronal Cells

**Figure 6:**
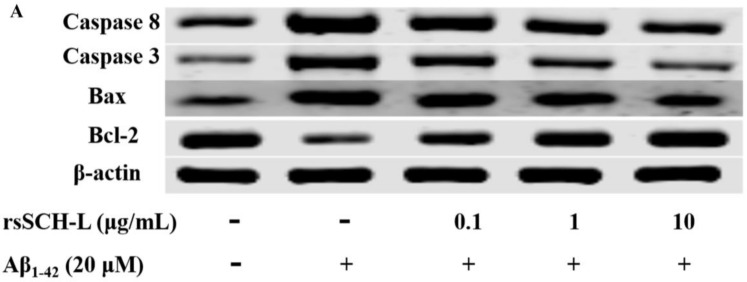

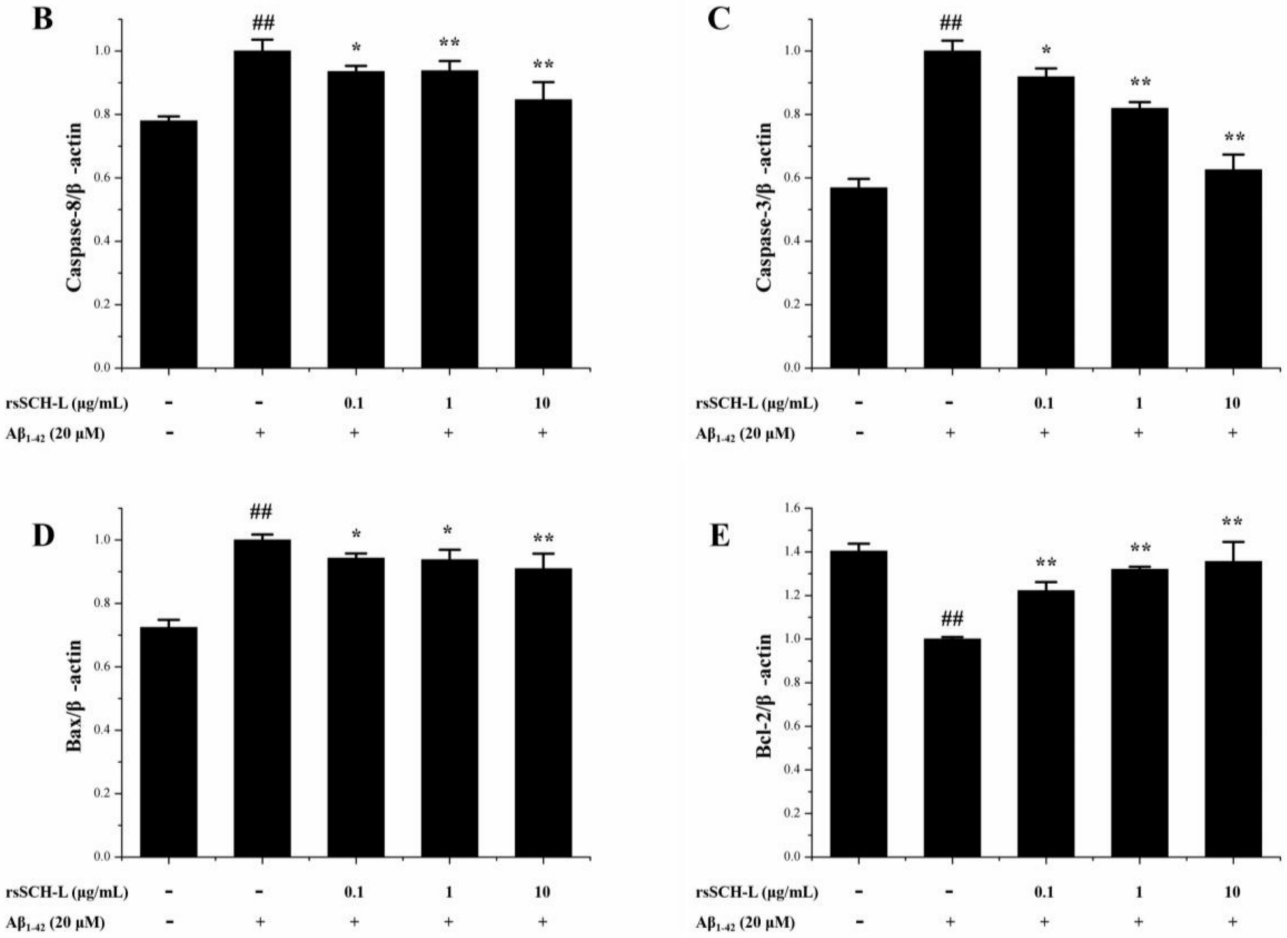
Effects of rsSCH-L on the expressions of Caspase-8, Caspase-3, Bax, and Bcl-2 proteins in neuronal cells. (**A**) Caspase-8, Caspase-3, Bax, and Bcl-2 protein expressions, (**B**) Caspase-8 protein level in neuronal cells, (**C**) Caspase-3 protein level in neuronal cells, (**D**) Bax protein level in neuronal cells, and (**E**) Bcl-2 protein level in neuronal cells. ^##^ *p* < 0.01 vs. control group, * *p* < 0.05, ** *p* < 0.01 vs. Aβ_1-42_ group.

To explore the apoptosis mechanism of the neuroprotective effect of rsSCH-L, the expressions of Bax, Bcl-2, and caspase-related proteins were investigated. Compared with the control group, the expressions of Caspase-8, Caspase-3, and Bax in the model group were increased while the expression of Bcl-2 was decreased. In the rsSCH-L treated group, the tendencies of the protein expression were reversed in a concentration-dependent manner (see Figure 6). These findings indicated that rsSCH-L possessed an inhibitory effect on Aβ_1-42_-induced apoptosis.

## 3. Discussion

Oxidative stress can cause neuronal damage and exacerbate senile degenerative diseases [32]. The current medicine cannot treat AD effectively since some drugs only temporarily improve the condition and lead to severe side effects [13,33]. In this circumstance, searching for potential protective agents in natural products and reducing oxidative stress-induced neurotoxicity may be helpful in preventing and treating neurodegenerative diseases.

In vivo studies showed that rsSCH-L significantly ameliorated cognitive memory impairment in the Aβ_1-42_-induced AD rat model. The space exploration experiment demonstrated that the cross platform times in high dose group and low dose group were more than those in the model group. Therefore, these results indicated that rsSCH-L owned the ability to improve AD learning and memory in rats.

Since rsSCH-L could significantly improve memory impairment in vivo, an in vitro experiment was conducted to explore the mechanisms of rsSCH-L on neuronal cells. In this experiment, we used the Aβ-induced primary neuronal cell model to study the neuroprotective effects of rsSCH-L. Primary neuronal cells usually maintain the basic nature of the original cells. This is widely used in exploring the mechanism of neuroprotection [11,34,35]. Aβ deposition has been confirmed as a pathological feature of AD. Aβ possessed the effect of oxidative stress and has been used to induce neuronal cell damage in many research studies [36,37]. Therefore, we used Aβ as an oxidative stress inducer to simulate the neuronal cell AD model and investigate the effects of rsSCH-L.

Our study showed that rsSCH-L could protect neurons from Aβ-induced damage. Aβ could increase the levels of LDH, MDA, and ROS as well as decrease the activities of SOD and GSH-Px. LDH is an enzyme present in the cytoplasm. When the cell membrane is damaged, LDH can be released [38]. MDA, which is a major product of lipid peroxidation, can directly reflect the extent of cell membrane damage [39]. Excessive ROS can produce potent oxidative stress [40]. SOD and GSH-Px are the major antioxidant enzymes that protect cells from injury [41]. rsSCH-L increased cell viability in a concentration-dependent manner. In addition, rsSCH-L reduced the levels of LDH, MDA, and ROS while the activities of SOD and GSH-Px were enhanced. The protective effect of rsSCH-L might be due to its antioxidant activity.

rsSCH-L can significantly inhibit neuronal cell apoptosis, which results from oxidative stress. Therefore, in our study, the levels of Caspase-3, Caspase-8, Bax, and Bcl-2 were further confirmed by western blotting. Bcl-2 and Bax are mainly concentrated in mitochondria. Bax is a pro-apoptotic protein and Bcl-2 is an anti-apoptotic protein. Caspase-8 and Caspase-3 play central roles in the initiation and are perpetrators of caspase cascade, respectively [42,43,44]. Our data showed that rsSCH-L could reduce the Caspase-8, Caspase-3, and Bax expressions and increase Bcl-2 expression. These results indicated that the neuroprotective effect of rsSCH-L was involved in the apoptotic pathway.

## 4. Materials and Methods

### 4.1. Materials

Dulbecco’s modified Eagle medium (DMEM) was purchased from Corning Cellgro Inc. (Herndon, VA, USA) and the fetal bovine serum (FBS) was obtained from Biological Industries Technologies (Kibbutz Beit Haemek, Israel). The Aβ_1–42_ peptide, DMSO, and MTT were acquired from Sigma-Aldrich (St Louis, MO, USA). The Aβ_1-42_ was dissolved in physiological saline and diluted to the concentration of 1 mg/mL. In order to get the oligomeric form for the cell tests, the Aβ_1–42_ solution was incubated at 4 °C for 24 h and the condition of 37 °C for 5 days was set to obtain agglomerative form for in vivo tests [11,21]. Hoechst 33258, Trypsin-EDTA solution, and penicillin-streptomycin solution were obtained from Beyotime Biotechnology Co. Ltd. (Shanghai, China). Neurobasal medium, B-27 and GlutaMAX supplements were obtained from Gibco Technologies (Grand Island, NY, USA). The Caspase 3 (H-277), Bax (P-19), and Bcl-2 (N-19) primary antibodies were acquired from Santa Cruz Biotechnology Inc. (Dallas, TX, USA). The β-III Tubulin, Goat Anti-rabbit IgG/RBITC, Caspase 8, and β-actin were purchased from Bioss Biotechnology Co. Ltd. (Beijing, China).

### 4.2. Preparation of rsSCH-L

The rattan stems of *S. chinensis* was collected from Raohe county in China. The plant materials were identified by associate professor Ruifeng Fan (Department of Chinese Medicine Resources, Heilongjiang University of Chinese Medicine). Briefly, *Schisandra chinensis* rattan stems were extracted for three times with 95% ethanol and each time for 2 h. Afterward, the crude extracts were eluted with 50% ethanol and 95% ethanol on an HPD-100 macroporous resin column and the 95% ethanol fractions were collected as the lignan fractions [27].

### 4.3. Animals

Eighteen-month-old adult Sprague-Dawley rats weighting 330–370 g (half male and female) were used for in vivo experiments (Certificate: SCXK2016-0005). All experimental animals were approved by the Institutional Ethics Committee of Heilongjiang University of Chinese Medicine. Fifty rats were divided into five groups randomly including a control group, model group (Aβ_1–42_), the Donepezil (Aβ_1–42_ + 2 mg/kg group), the rsSCH-L 200 mg/kg treated group (Aβ_1–42_ + 200 mg/kg rsSCH-L), and the rsSCH-L 66.67 mg/kg treated group (Aβ_1–42_ + 66.67 mg/kg rsSCH-L). In addition to the control group, other groups were anesthetized with chloral hydrate and then Aβ_1-42_ peptide was injected into left and right ventricles (AP, −2.8 mm, ML, ±1.9 mm, DV, −4.2 mm). The Aβ_1-42_ with the volume of 5 µL was injected in 5 min. The needle was slowly removed in the fifth minute after injection and the incision was sutured [11,21]. Starting the next day, rats of each group were administered corresponding drug daily for 30 consecutive days by intragastric infusion.

### 4.4. Morris Water Maze Test

The Morris water maze test was carried out to evaluate the learning and memory abilities in rats [45,46,47]. Acute training of rats lasted for 5 consecutive days starting on the 26th day. The spatial exploration experiment was conducted in a circular pool (150 cm in diameter, 50 cm in height, and 30 cm in depth) on the 30th day. The pool was divided into 4 quadrants and the platform was placed 1 cm underwater in the fourth quadrant. The rat was released in the second quadrant. The swimming trajectory and time to the forth quadrant was recorded. If the rat did not find the platform within 60 s, the rat was removed to the platform for 20 s. In the last training trial, the platform was removed and the number of mice that passed the platform was recorded within 60 s. All the experiments were performed with video tracking equipment for data acquisition and analysis by using behavioral analysis software (Techman soft, WMT-100, Chengdu, China).

### 4.5. Primary Neuronal Cells Culture

Primary cortical neuronal cells were isolated from Sprague-Dawley rat pups (The animal approval number is: SYXK HEI 2016-015, which was approved by the Animal Care Committee of School of Medicine, Heilongjiang University of Chinese Medicine). The cortical tissues were separated and then cut into small pieces. The pieces were digested with 0.125% trypsin for 20 min at 37 °C. Digestion was stopped with DMEM. A 70 μm filter membrane was used to filter the cells. The suspension was added to PDL-coated 6-well plates for 4 h. The medium was changed to neurobasal medium containing B27 and GlutaMAX supplement. After seven days, the cells were prepared for further study [35].

### 4.6. Immunofluorescence Identification

The purified neuronal cells were seeded on Poly-D-lysine-treated 6-well plates and then fixed with 4% PFA for 20 min. The cells were incubated with 0.3% Triton X-100 for 20 min and 10% sheep serum for 30 min, primary antibody β-III Tubulin (1:200) overnight at 4 °C. This was followed by a secondary Goat Anti-rabbit IgG/RBITC (1:500) 37 °C for 2 h. The cells were stained with 5 mg/mL Hoechst 33258 for 30 min in the dark, then washed with PBS three times, and imaged by using a fluorescence microscope (Zeiss, Axio Vert.A1, Oberkochen, Germany) [48].

### 4.7. MTT Assay

The cell viability was measured by using the MTT assay. Primary neuronal cells were seeded at a density of 1 × 10^4^ cells/well and were allowed to attach for 24 h in 96-well plates. After incubation with rsSCL-L (0.1, 1 and 10 μg/mL) and Aβ_1-42_ (20 μM) for 24 h, the cells were added with 10 μL MTT (5 mg/mL) and incubated for 4 h at 37 °C. Subsequently, the medium was carefully removed and the cells were dissolved in dimethyl sulfoxide (DMSO) for 10 min. The absorbance was measured at 490 nm with a multi-detection microplate reader (PerkinElmer, VICTOR X3, Waltham, MA, USA).

### 4.8. LDH Assay

The level of LDH was used to determine cell cytotoxicity. The culture supernatants were collected and accumulation of LDH was detected using a commercial assay kit (Nanjing Jiancheng Bioengineering Institute, Nanjing, China). The absorbance was measured at 440 nm with a microplate spectrophotometer (BioTek, Epoch2, Winooski, VT, USA).

### 4.9. Assays of Oxidative Stress

After incubation with rsSCL-L (0.1, 1 and 10 μg/mL) and Aβ_1-42_ (20 μM) for 24 h, the cells were collected. The levels of ROS, MDA, SOD, and GSH-Px were detected using the commercial kits (Nanjing jiancheng Bioengineering Institute, Nanjing, China).

### 4.10. Quantification of Apoptosis

Primary cortical neuronal cells were centrifuged at 1000 rpm for 5 min to collect the cells. The cells were washed twice with PBS and then were re-suspended in Annexin V-FITC binding buffer. The cells were added with 5 μL Annexin V-FITC and 10 μL of PI staining solution. The cells were incubated at 25 °C for 15 min in the dark room and then were determined by flow cytometry analysis (BD Accuri^TM^ C6, Becton, Franklin Lakes, NJ, USA).

### 4.11. Western Blot Assay

Neuronal cells were cultured in 60 mm dish. After being incubated with rsSCL-L and Aβ_1-42_ for 24 h, the cells were collected and determined by using the BCA assay kit (Beyotime, Suzhou, China). Afterward, an equal amount of protein buffer was separated by 12% SDS-PAGE gels and was transferred to 0.22 μm NC membranes. The membranes were blocked with 5% (*w/v*) non-fat milk in PBST for 2 h. All the primary antibodies were incubated at 4 °C overnight. After being washed with PBST, the membranes were incubated with the secondary anti-rabbit antibody or anti-mouse antibody at room temperature for 1 h. After being washed with PBST, the ECL kit (Beyotime, Suzhou, China) was applied to develop the protein bands. The bands were scanned by using the molecular imager (BIO-RAD, Hercules, CA, USA).

### 4.12. Statistical Analysis

Statistical analyses were conducted with the Origin 8.0 software. All data were given as the mean ± standard deviation of at least three experiments. Values were compared using one-way analysis of variance (ANOVA) and the Dunnett’s test. *p* values less than 0.05 were accepted as statistically significant.

## 5. Conclusions

In summary, this research provides the first in vivo and in vitro evidence that lignans from *Schisandra chinensis* rattan stems (rsSCH-L) can protect the neuronal cells against Aβ_1-42_ through apoptosis and antioxidant activity. This study can promote the research of rsSCH-L for the complementary and alternative medicine to treat AD.
